# Development of a Quantitative Methodology to Assess the Impacts of Urban Transport Interventions and Related Noise on Well-Being

**DOI:** 10.3390/ijerph120605792

**Published:** 2015-05-26

**Authors:** Matthias Braubach, Myriam Tobollik, Pierpaolo Mudu, Rosemary Hiscock, Dimitris Chapizanis, Denis A. Sarigiannis, Menno Keuken, Laura Perez, Marco Martuzzi

**Affiliations:** 1 European Centre for Environment and Health, World Health Organization (WHO) Regional Office for Europe, Platz der Vereinten Nationen 1, 53113 Bonn, Germany; E-Mails: mudup@ecehbonn.euro.who.int (P.M.); martuzzim@ecehbonn.euro.who.int (M.M.); 2 Federal Environment Agency, Section II 1.6 Exposure Assessment and Environmental Health Indicators, 14195 Berlin, Germany; E-Mail: myriam.tobollik@uba.de; 3 School of Geographical Sciences, University of Bristol, University Road, Bristol BS8 1SS, UK; E-Mail: r.hiscock@bristol.ac.uk; 4 Department of Chemical Engineering, Aristotle University of Thessaloniki, Environmental Engineering Laboratory, 54124 Thessaloniki, Greece; E-Mails: dimitris.chapizanis@gmail.com (D.C.); denis@eng.auth.gr (D.A.S.); 5 Netherlands Organisation for Applied Scientific Research (TNO), 3508 TA Utrecht, The Netherlands; E-Mail: dimitris.chapizanis@gmail.com; 6 Swiss Tropical and Public Health Institute, Socinstr. 57, 4002 Basel, Switzerland; E-Mail: l.perez@unibas.ch; 7 University of Basel, Peterspl. 1, 4003 Basel, Switzerland

**Keywords:** urban policies, climate change, mitigation, greenhouse gas, transport, noise, well-being, impact assessment

## Abstract

Well-being impact assessments of urban interventions are a difficult challenge, as there is no agreed methodology and scarce evidence on the relationship between environmental conditions and well-being. The European Union (EU) project “Urban Reduction of Greenhouse Gas Emissions in China and Europe” (URGENCHE) explored a methodological approach to assess traffic noise-related well-being impacts of transport interventions in three European cities (Basel, Rotterdam and Thessaloniki) linking modeled traffic noise reduction effects with survey data indicating noise-well-being associations. Local noise models showed a reduction of high traffic noise levels in all cities as a result of different urban interventions. Survey data indicated that perception of high noise levels was associated with lower probability of well-being. Connecting the local noise exposure profiles with the noise-well-being associations suggests that the urban transport interventions may have a marginal but positive effect on population well-being. This paper also provides insight into the methodological challenges of well-being assessments and highlights the range of limitations arising from the current lack of reliable evidence on environmental conditions and well-being. Due to these limitations, the results should be interpreted with caution.

## 1. Introduction

Cities produce approximately 75% of the global carbon emissions [1] and therefore are in a unique position to facilitate effective reductions of greenhouse gas (GHG) emissions. The European Union (EU) project “Urban Reduction of Greenhouse Gas Emissions in China and Europe” (URGENCHE) aimed to quantify health and well-being co-benefits of urban policies targeted at greenhouse gas emission reduction as required by the Kyoto Protocol commitment for a 20% GHG reduction by 2020 [2,3]. The urban policies to be evaluated concerning their co-benefits were provided by seven project cities (Basel (Switzerland), Kuopio (Finland), Rotterdam (the Netherlands), Stuttgart (Germany), Suzhou (China), Thessaloniki (Greece) and Xi’an (China)) and relied on the real planning in the cities, making the results highly relevant for local decision-making. The URGENCHE project framework considered three policy areas which are largely influenced by local authorities and affect both climate and GHG emissions: energy production and distribution (e.g., use of renewables), transport (e.g., modal split, traffic reduction, electric cars), and housing (e.g., energy efficiency) [4,5]. 

For the assessment of health impacts of the urban interventions, HIA approaches were applied within URGENCHE to quantify impacts of particularly transport policies and related interventions on levels of air pollution, noise and physical activity and their ensuing implications for health [6,7]. For these HIA, standardized approaches and risk estimates derived from validated exposure response functions between environmental conditions and selected health outcomes can be applied [8,9,10]. However, there are no standard approaches or risk estimates available to describe the well-being impacts of environmental features; instead there is ongoing debate on the conceptualisation and measurement of well-being [5,11,12,13,14,15,16]. In the absence of a standardized approach on well-being impact assessment, and lacking a consensus-based definition of well-being, alternative mechanisms to define and assess the well-being impact of urban interventions were explored. 

For the purposes of URGENCHE, we developed a conceptualization of well-being that reflects a subjective approach and is measured through assessing individual’s self-perceptions of their well-being [13,15], rather than a more objective construction of well-being using indicators such as e.g., income or objective living conditions [17,18]. There are many approaches to define subjective well-being and due to different epistemologies and schools of thought, diverse well-being measurement tools have been suggested [5,12,13,14,19,20,21]. Yet, the theoretical and empirical literature strongly supported the notion that subjective well-being includes both hedonic dimensions (emotions and feelings in relation to pleasure and happiness) and eudemonic dimensions (perception of people’s functioning in relation to self-esteem, autonomy and fulfilment) [5,15,20,22]. Subjective well-being therefore goes beyond the objective and evaluative well-being that judges certain conditions and, in environmental terms, leads to complaints or similar indications of annoyance or disturbance which are strongly associated with a specific condition but may have a less strong impact on overall subjective well-being. Based on this understanding of subjective well-being, we gave preference to multi-faceted well-being measurement tools which are based on a number of items (rather than a single question) and incorporated measures of hedonism as well as eudemonism. 

For the well-being impact assessment of urban policies for the URGENCHE project, focus was put on transport interventions which may affect urban environments through potential changes in air pollution and noise emission. Traffic noise was chosen for the assessment of well-being impacts as noise perception is more likely to affect subjective well-being than air pollution (which may be more difficult to be assessed by individual residents unless pollution levels are very high). The selection of noise also acknowledges that road traffic noise is considered the most widespread urban noise source in Europe, and a rising urban challenge associated with well-being [23]. In summary, the project aimed to explore:

(1) if existing information on environmental exposure, environmental perception and well-being can be applied and linked to derive a first indicative assessment of the potential well-being benefits of urban climate mitigation interventions, and
(2) which methodological challenges and limitations arise, indicating and discussing areas of uncertainty due to lack of evidence and validated assessment procedures.

This paper presents the results and lessons learned of this first exploratory attempt to quantify noise-related well-being impacts of urban transport interventions at population level. 

## 2. Data and Methods

For assessing the impact of local transport interventions and the related traffic noise exposure changes on well-being, data on noise exposure before and after the intervention are needed. This was possible for Basel (Switzerland), Rotterdam (The Netherlands) and Thessaloniki (Greece), while Stuttgart (Germany) was not able to provide noise modelling for the planned intervention scenario. In Kuopio (Finland) and the two Chinese cities of Suzhou and Xi’an, the planned transport interventions had no impact on noise as they focused on fuel use and reduction of emissions. The year 2010 was selected as the starting point of the assessment (Baseline2010) and 2020 as the point in time when the effects of the intervention will be measured (Intervention2020). To account for other changes occurring between 2010 and 2020, a Business-as-Usual scenario (BAU2020) was established, including changes and developments from 2010 to 2020 that are independent of the planned intervention. 

### 2.1. Data: City Interventions

The transport-related interventions affecting noise exposure are described in Table 1 together with the respective Business-as-Usual scenarios).

**Table 1:** Interventions to be implemented in case study cities by 2020.

**Basel**	A scenario was developed by the city that accounts for additional local transport measures beyond a Business-as-Usual scenario (BAU2020) to further reduce private car road traffic by 4% on inner roads. It includes traffic measures targeted at channelling traffic along main avenues, reducing traffic levels and enforcing moderate speed limits in residential areas. The BAU2020 scenario in Basel does already include a range of measures adopted by the city by 2010 and implemented before 2020 (tram line extensions, expanding t capacity of main highways). An overall increase of 8% of vehicle kilometres has been estimated by 2020.
**Rotterdam**	50% of local car fleet will be electric cars (excluding motorbikes, vans and trucks). The BAU2020 scenario does not include specific interventions but accounts for expected changes in fleet composition related to Euro emission classes. It assumes zero growth of the traffic volume at inner-urban roads and 2% growth of the traffic volume at motorways as compared to the Baseline 2010 situation.
**Thessaloniki**	A local metro built in central Thessaloniki will reduce private road transport by an expected 33%‒44% in the city center and by 22% on main road axes leading to suburbs. The Intervention2020 scenario also includes a higher share of diesel and hybrid, but only a small (2%) share of electric vehicles in the fleet compared to the Baseline 2010. The BAU2020 scenario does not include specific interventions and serves as an extrapolation of the Baseline 2010 situation.

### 2.2. Data for Well-being Impact Assessments of Urban Interventions

Transport interventions for GHG reduction purposes are also expected to lead to reductions in traffic noise exposure, which may have a positive effect on well-being. The change of overall well-being of the urban population can be quantified by assessing well-being before and after the interventions, indicating to what extent the population will benefit from the urban GHG reduction measures in terms of well-being. For such an assessment, data are required for (a) traffic noise exposure before and after the intervention; and (b) well-being impacts of traffic noise exposure.

#### 2.2.1. Data on Road Traffic Noise Exposure on City Level

Changes in traffic noise exposure were calculated comparing the noise levels of the Intervention2020 and BAU2020 scenarios to the noise levels in the 2010 baseline scenarios. Noise level data were obtained from locally developed models for each city, using the respective standard methodology applied for noise mapping and modeling (see Supplementary File 1 for details). Data were provided in the format of Lden at façade or building entrance points. Lden is a weighted noise average over 24 h, assigning higher weights to the evening and night periods than to the day period (following European noise regulation [24]). For all cities and scenarios, the fraction of population exposure by 1dB Lden was calculated. The restrictions of using city models on traffic noise based on Lden are discussed in Section 4.2 on limitations.

#### 2.2.2. Data on Well-Being Impacts of Traffic Noise

A review of literature identified only four studies which suggest a quantitative association on road traffic noise and well-being (Table 2), but this evidence is strongly limited and mostly based on local studies or specific contexts. Only two of the four studies involved a larger sample size, one study focused on a very specific setting (rural alpine communities affected by through-traffic noise), and one study was restricted to one city only. All studies were cross-sectional and only two included control variables in the analysis. Finally, two out of four studies did not provide significant results on well-being impacts of road traffic noise. Based on these studies, no generally applicable risk ratio or exposure-response function between noise and well-being could be derived.

Other noise-specific studies providing quantitative risk ratios were identified, but these focused on more health-specific outcomes such as mental health or depression [25,26,27,28,29,30], or health-related quality of life [31,32,33,34]. As well, some studies referred to well-being-related impacts of e.g., aircraft noise or railways [28,35,36,37,38,39] but these were not considered applicable for road traffic. A range of noise studies provide dose-response relations between transport noise levels and noise annoyance which sometimes are highly significant [40,41,42,43,44,45,46,47], but as noise annoyance mainly contributes to evaluative and objective well-being, it may not adequately reflect the multi-faceted concept of general subjective well-being [15,46,47]. Also, noise annoyance is often used in HIA as an independent health outcome and URGENCHE-related HIA work on transport interventions has also applied annoyance accordingly [7,48]. Annoyance was therefore not considered to fully reflect the concept of well-being and instead, preference was given to validated well-being tools or at least more generic indicators of general life satisfaction (for more discussion on well-being assessment approaches, see [5,13,14,15,20]). Challenges associated with the operationalization of “well-being” are discussed in Section 4.2 on limitations.

Hiscock *et al*. have indicated in this context that risk ratios for noise and well-being are rare mainly because of the following points: insufficient quality and quantity of available studies; diversity of measures on well-being outcomes; and the rather varying risk ratios indicated by the studies (for strongly different population groups in rather different local contexts) [5]. Also, it must be noted that any environmental variable is likely to have a less strong impact on subjective well-being measured by multidimensional tools (which are more robust vis a vis the potential influence of individual aspects) than on well-being measured through cause-specific evaluative approaches (such as annoyance or complaints). During our literature review, we also noted that there is mixed evidence regarding the equity dimension of noise exposure or annoyance, with some studies pointing to increased noise exposure in disadvantaged population groups [49,50,51] and some studies not finding such disadvantage [45,52,53], but no study looking at well-being impacts of noise exposure from an equity lens. 

**Table 2:** Papers presenting quantitative associations between noise and well-being measures.

Author	Noise Measure	Well-being Measure	Setting	Study Design/Sample Size	Controls	Association	Significant	Restriction
Lercher/Kofler, 1996 [40]	Noise level from traffic	Loss of well-being; Life satisfaction	Five rural alpine communities, Austria	Cross-sectional; n = 1989	Age, Sex, SES	Loss of well-being (dichotomized) OR 1.50 (1.14‒1.96) above 55 decibels *vs*. 55 or lower; Life satisfaction (dichotomized) OR 0.68 (0.51‒0.90) above 55 decibels *vs*. 55 or lower	Yes	Specific setting unlikely to reflect urban noise conditions
Schreckenberg *et al*., 2010 [28]	Daytime noise level (road)	Life satisfaction (FLZ score)	Frankfurt, Germany	Cross-sectional, n = 190	None	Life satisfaction coefficient of correlation = 0.103	No	Small sample, one city only
Urban/Maca, 2013 [38]	Road noise (strategic noise maps)	Life satisfaction	5 Czech cities	Cross-sectional; n = 354	None	Life satisfaction r = 0.066	No	Small sample, Czech data only
Rehdanz/Maddison, 2008 [54]	Perceived local noise nuisance	Life satisfaction	Germany	Cross sectional; n = 23,000	Unclear	For each step reduction in feeling adversely affected by noise (5 steps from not at all to very strongly), a person is 0.85% more likely to score highest life satisfaction levels, 0.63% less likely to score average life satisfaction levels, and 0.34% less likely to score lowest life satisfaction levels.	Yes	German environmental preference data only

As no local data on traffic noise and well-being associations were identified for the three cities, and no studies were identified that enabled the valid derivation of well-being effects of traffic noise, national survey data covering overall noise perception and a well-being score were therefore used as the best available proxy instead. 

Noise and well-being data applied for Rotterdam and Thessaloniki were taken from the European Quality of Life Survey 2012 (EQLS2012) [15]. EQLS2012 data cover both perceived noise exposure (no, moderate and major noise problems) and the internationally validated WHO_5 well-being index [55,56], enabling the quantification of associations between noise perception and well-being. The WHO_5 includes five items covering both hedonic and eudemonic aspects of well-being (ability to relax, feeling rested, cheerfulness and good spirits, being active, experiencing life as full of interest, and results in a score from 0 to 100. We chose a cut-off of 52 to represent a binary outcome of lower *versus* higher well-being as suggested elsewhere [19,55,56]. As Switzerland did not participate in the EQLS, data for Basel were taken from wave 14 of the 2012 Swiss Household Panel (SHP2012) [57]. The SHP2012 includes questions on noise annoyance with a dichotomous response (yes/no) and self-assessed mental well-being on a scale from 0 (never) to 10 (always); a score ≥ 6 was chosen as the cut-off to characterize individuals with low well-being. Both EQLS2012 and SHP2012 include relevant confounders of the noise-well-being association (such as age, gender, income, education), and allow rural residents to be identified and excluded from analysis. Table 3 provides an overview of the data components selected for the well-being assessment. 

**Table 3:** Data used for the well-being impact assessment.

City	Urban Noise Exposure Changes	Association between Urban Noise Perception and Well-Being		
		Data Source	Noise Variable	Well-Being Variable
**Basel**	Local noise models (Lden)	Swiss Household Panel 2011, urban residents (n = 4505)	Annoyed by noise from neighbours or noise from the street (traffic, business, factories *etc*.). (Yes—No)	Do you often have negative feelings such as having the blues, being desperate, suffering from anxiety or depression? (Scale from 0 = “never” to 10 = “always”)
**Rotterdam**		EQLS2012, Dutch urban residents (n = 582)	Thinking of your immediate neighbor-hood—do you have problems with noise? (Major problems—Moderate problems—No problems)	WHO_5 well-being index (5 items producing a scale from 0 to 100)
**Thessaloniki**		EQLS2012, Greek urban residents (n = 631)		

### 2.3. Methods for Well-Being Impact Assessment 

Step 1—Derivation of noise-well-being association using national proxy data

To avoid influence from rural noise conditions, each national dataset was reduced to urban-only residents. Differences between national and urban-only data were rather modest for age and gender as well as for noise and well-being levels, except for Greece where noise problems are more frequently reported in urban areas (see Supplementary File 2, Table S1). Similarly to previously published studies, the well-being variable was dichotomized and used as the dependent variable in logistic regression models [18,40,54]. As independent variables, the models included several covariates taken from the EQLS and SHP datasets, such as gender, age, income, education, employment, making ends meet financially and household structure (see Supplementary File 3, Table S2). For each regression model, the standardized residuals were checked for outliers which were deleted and the regression repeated. 

Standardized residuals, group membership and predicted well-being probability were saved (similar to the approach by Rehdanz and Maddison 1998 [54]), using a 0.5 cut-off [58]. This creates a variable indicating the predicted classification of high *versus* low well-being based on the covariate characteristics of the individuals, which can be compared with the observed well-being classification based on the WHO_5 well-being index. This comparison is reflected by the model classification tables that indicate how much the model correctly classifies the cases. The overall prediction of the final regression models for the total urban sample was 75.8% in Greece (n = 425), 81.9% in the Netherlands (n = 487), and 91.8% in urban Switzerland (n = 4505) (see Supplementary File 4, Table S3, for details).

The predicted well-being probability score covers a range from 0 (indicating the lowest probability of well-being) to 1, indicating the highest probability of well-being after taking into account the respective covariates [59]. The well-being probability score can also be expressed in %.

Mean values of predicted well-being probability were calculated for the noise perception categories (no, moderate and major noise problems in Greece and the Netherlands and noise annoyance *versus* no noise annoyance in Switzerland) to define the well-being probability of the subpopulation in the respective noise perception category. These predicted probability values for the noise categories represent the “noise-well-being-relationship” that is later-on used for the well-being assessment of the URGENCHE interventions in the cities. 

Step 2—Transfer of EQLS and SHP noise perception to local traffic noise models for case study cities

The well-being effect of the urban transport interventions is assessed by combining the modeled city data on traffic noise exposure changes with the noise-related well-being probability (derived from the EQLS2012 and SHP2012 data). The combination is based on the assumption that EQLS2012 and SHP2012 data indicate the population percentage complaining about noise problems, and this percentage is applied to define the Lden ranges associated with certain noise perception categories in the respective city noise models (see details in Supplementary File 5, Table S4). The connection of local traffic noise exposure data with the noise perception data from EQLS2012 and SHP2012 results in cut-offs for low, medium and high noise perception tabled below which are slightly different in each city (Table 4). 

**Table 4:** Noise perception category ranges for the city noise exposure profiles.

Noise Perception	Basel	Noise Perception	Thessaloniki	Rotterdam
**High: Annoyed by noise**	≥64 dB	**High: Major problems**	≥65 dB Lden	≥67.5 dB Lden
*	*	**Medium: Moderate problems**	55‒64 dB Lden	57.5‒67.4 dB Lden
**Low: Not annoyed by noise**	<64 dB	**Low: No problems**	≤54 dB Lden	<57.5 dB Lden

* The SHP2011 dataset used for Basel does only distinguish between the perception of a noise as annoying or not annoying. It did not offer an intermediate option.

Traffic noise exposure changes in the different city scenarios lead to a changed percentage of the population predicted to report high, medium or low noise perception in the 2020 scenarios. As each noise perception category is associated with a specific probability of well-being, changes in population well-being can be finally estimated for each case study city and scenario. 

The use of EQLS2012 and SHP2012 data introduces a strong limitation as it links well-being levels associated with the overall perception of urban noise (taken from the surveys) with traffic noise data provided by the cities (see further discussion in Section 4.2 on limitations). 

Step 3—Exploratory well-being impact assessments for the less affluent population

As research and political attention has increasingly focused on equity effects of public health and environmental interventions, well-being probability predictor values were also produced for the less affluent city population (defined as the two lowest income quartiles). Based on these values, we repeated the well-being impact assessment of noise reduction effects triggered by the urban interventions for the less affluent populations in each city. 

The overall prediction of the final regression models for the urban sample of less affluent population groups was 73.9% in Greece (n = 199), 79.6% in the Netherlands (n = 245), and 89.6% in urban Switzerland (n = 2253) (see Supplementary File 4, Table S3 for details). 

## 3. Results

### 3.1. Traffic Noise Exposure Changes Associated with the Interventions

While the noise model predicts almost no traffic noise variations between the scenarios in Rotterdam, there is a marked reduction of population exposure to noise by the Intervention2020 scenario in Thessaloniki. In Basel, the BAU2020 provides a strong reduction of high noise levels while the Intervention2020 scenario actually increases again the prevalence of high noise levels (see Supplementary File 6, Figure S1−S3 for details). The modeled change of the population percentage categorized into different noise perception categories is shown in Table 5. 

**Table 5:** Changes of perceived noise exposure in the case study cities.

**Level of Perceived Noise Exposure in BASEL**	**Population Exposed in Baseline2010**	**Population Exposed in BAU2020**	**Population Exposed in Intervention2020**
	**Total Population**	**Total Population**	**Total Population**
**High: Annoyance by noise (≥64 dB)**	19.8%	10.8%	13.9%
**Low: No annoyance by noise (<64 dB)**	80.2%	89.2%	86.1%
**Level of Perceived Noise Exposure in ROTTERDAM**	**Population Exposed in Baseline2010**	**Population Exposed in BAU2020**	**Population Exposed in Intervention2020**
	**Total Population**	**Total Population**	**Total Population**
**High: Major noise problem ((≥67.5 dB)**	1.6%	1.9%	1.7%
**Medium: Moderate noise problem (57.5−67.4 dB)**	19.5%	20.4%	19.7%
**Low: No noise problem (≤57.4 dB)**	78.9%	77.6%	78.6%
**Level of Perceived Noise Exposure in THESSALONIKI**	**Population Exposed in Baseline2010**	**Population Exposed in BAU2020**	**Population Exposed in Intervention2020**
	**Total Population**	**Total Population**	**Total Population**
**High: Major noise problem (≥65 dB)**	15.2%	15.4%	8.9%
**Medium: Moderate noise problem (55-64.9dB)**	40.6%	40.5%	36.4%
**Low: No noise problem (≤54.9dB)**	44.2%	44.1%	54.7%

HIA work on noise effects of urban interventions based on known dose-response functions between noise and noise annoyance suggests that, reflecting the noise reductions in each city, the transport interventions cause a slight reduction of strong noise annoyance [7,48].

### 3.2. Well-Being Probability Predictors

The well-being probability predictor values are shown in Table 6 for each city and noise perception category. The well-being probability of the total population differs strongly between the cities, with the lowest probability found in Thessaloniki (63.1%) and the highest probability found in Basel (91.8%). 

For all cities, the lowest well-being probability is found for the population group reporting high levels of noise. However, only in Rotterdam there is a straight dose-response relationship indicating that less noise is associated with a higher well-being probability, while in Thessaloniki the highest well-being probability is actually found for the persons reporting moderate noise problems (although the difference between low and medium noise perception is marginal). In Basel, the effect of high noise perception on well-being is less strong than in Rotterdam or Thessaloniki. 

**Table 6:** Well-being probability in relation to noise perception.

Level of Perceived Noise Exposure	Predicted Well-Being Probability (in %)
**BASEL**	**Total Urban Population Sample (n = 4505)**
**High (≥64 dB)**	89.8%
**Low (<64 dB)**	92.4%
**Total population**	91.8%
**ROTTERDAM**	**Total Urban Population Sample (n = 487)**
**High (≥67.5 dB)**	73.1%
**Medium (57.5‒67.4 dB)**	78.1%
**Low (≤57.4 dB)**	80.0%
**Total population**	79.5%
**THESSALONIKI**	**Total Urban Population Sample (n = 424)**
**High (≥65 dB)**	55.9%
**Medium (55‒64.9 dB)**	64.7%
**Low (≤54.9 dB)**	64.1%
**Total population**	63.1%

### 3.3. Well-Being Assessment at Urban Level

Combining the changes in noise levels with the respective well-being probability for each city and scenario provides indications of well-being changes for the total city population (Table 7, Table 8 and Table 9). As only a small proportion of the population moves from high to medium or low noise perception, and the well-being probability increase is limited as well, the overall well-being changes tend to be marginal on population level. More pronounced well-being impacts are therefore only found in residents reporting high noise perception at baseline.

For the total population of Basel, the well-being probability only increases by 0.3% from 91.8% at Baseline2010 to 92.1% under Intervention2020. Yet, the BAU2020 already achieved a well-being probability of 92.1%, indicating that the Intervention2020 scenario provides no additional impact (Table 7). Compared to the total population, a stronger well-being impact is found for the residents reporting high noise levels (increase of 0.8%). However, for those residents reporting high perceived noise levels, the BAU2020 was even more effective (probability of well-being increase by 1.2%) but this effect is partially lost in the Intervention2020 scenario. 

**Table 7:** Well-being probability in Basel by noise levels.

Basel	Intervention Implemented by 2020	Predicted Well-Being Probability (in %)		
		Baseline2010	BAU2020	Intervention2020
**Total population**	Local transport scenario Z9, reduction of traffic by 4% *(NB: BAU2020 also includes various transport measures)*	**91.8%**	**92.1%**	**92.1%**
High noise perception		89.8%	91.0%	90.6%
Low noise perception		92.4%	92.4%	92.4%

The Rotterdam results show a 50% electric local car fleet has no well-being impact for the local population (Table 8). The negligible change of well-being probability is caused by the fact that the population reporting high noise levels did not change much between the scenarios. Yet, for the 1.6% of the population reporting high noise perception at baseline, there is at least some increase in well-being probability.

**Table 8:** Well-being probability in Rotterdam by noise levels.

Rotterdam	Intervention Implemented by 2020	Predicted Well-Being Probability (in %)		
		Baseline2010	BAU2020	Intervention2020
**Total population**	50% of car fleet are electric cars	**79.5%**	**79.5%**	**79.5%**
High noise perception		73.1%	73.1%	73.8%
Medium noise perception		78.1%	78.1%	78.2%
Low noise perception		80.0%	80.0%	80.0%

In Thessaloniki, the well-being probability increases by 3.7% for residents reporting high noise levels (Table 9). Despite this relatively strong impact, the well-being increase remains limited for the total population because medium noise levels were associated with a slightly higher probability of well-being than low noise levels, leading to a decrease of overall well-being of the population in response to noise reduction for medium to low perception levels. This impact counteracts the increase of well-being probability found for residents reporting high noise exposure levels, and limits the overall well-being probability increase to 0.5% for the total population.

**Table 9:** Well-being probability in Thessaloniki by noise levels.

Thessaloniki	Intervention Implemented by 2020	Predicted Well-Being Probability (in %)		
		Baseline2010	BAU2020	Intervention2020
**Total population**	Local metro built in central Thessaloniki	**63.1%**	**63.1%**	**63.6%**
High noise perception		55.9%	55.9%	59.6%
Medium noise perception		64.7%	64.7%	64.6%
Low noise perception		64.1%	64.1%	64.1%

### 3.4. Well-Being Assessment for Less Affluent Population

The results of the well-being assessment for the less affluent population (two lowest income quartiles) are summarized below. Detailed result tables can be found in Supplementary File 7, Table S5−S7. 

In all three cities, the less affluent population reports high noise perception more frequently, yet the noise reduction effect of the BAU2020 and Intervention2020 scenarios seems to be a bit smaller than for the total population. The well-being probability is lower for the less affluent population in all cities, with the largest difference in Thessaloniki (52.0% for less affluent population *versus* 63.1% for total population). Residents reporting high noise perception have a lower well-being probability, as already observed in the total population.

Well-being probability changes follow the same pattern as for the total population. In Basel, there is a small well-being benefit for the Basel BAU2020 scenario (increase by 0.5%) rather than the Intervention2020 scenario, while in Thessaloniki the Intervention2020 scenario increased well-being probability by 0.7%. In Rotterdam, the intervention has no impact on well-being at all. 

Another similarity to the results for the total population is that the largest well-being increase is found for persons reporting high perceived noise at baseline; the increase of well-being probability found for Thessaloniki residents reporting high noise levels at baseline (4.0%) is the strongest well-being benefit identified in all three cities. However, compared to the total population, there is no indication of a generally stronger well-being benefit for the less affluent population in all three cities.

## 4. Discussion

This paper has presented an exploratory assessment of population-based well-being impacts of urban transport policies and their noise effects in absence of standardized risk ratios and local studies linking urban noise with the well-being of citizens. It proposed and tested a methodology that aims to make pragmatic use of the data available and derived exploratory results, triggering discussion on the potential impact of urban interventions on population well-being and highlighting the main challenges and limitations of well-being assessments in terms of data and methodology. Below, the findings and potential implications of the well-being assessment are shortly discussed but, given the range of limitations, emphasis is put on the reflection of the methodological restrictions of currently available data for well-being assessments.

### 4.1. Main Findings

The main findings of this exploratory well-being assessment can be summarized as below:

(1) The expected noise exposure changes resulting from the urban transport interventions are rather limited in all three cities.
(2) Well-being probability is consistently reduced by perception of high noise levels, although this varies a lot in the different cities.
(3) Across all three cities, the noise-related increase of well-being probability associated with the urban transport interventions is marginal. The strongest increase in well-being probability is found for Thessaloniki (0.5%).
(4) Larger well-being benefits are consistently found for population groups reporting high noise levels at baseline. This is valid in all cities (especially Thessaloniki) and for total and less affluent populations.
(5) Less affluent population groups do not seem to derive a stronger well-being benefit from the transport interventions than the total population.

The very modest impact of the interventions on population well-being seems to be conservative in light of some results that have been published before in relation to noise impacts on well-being (e.g., OR 1.50 for loss of well-being [40]), life satisfaction (e.g., OR 0.68 for life satisfaction for noise levels above 55 dB [40], or mental well-being (e.g., OR 1.34 for dissatisfaction with street noise [26] or OR 2.9 for depression in women exposed to road traffic noise above 70 dB [30]), and rather matches studies reporting a marginal effect on well-being or life satisfaction [28,38], mental health status [33] or subjective health and well-being [18]. 

There are various reasons to explain the limited well-being impacts of the transport interventions. One explanation is that urban transport interventions may provide a rather limited noise reduction effect (see also Stansfeld *et al*. on a road traffic intervention study [29]), which restricts their potential well-being benefit. The reduction of road traffic or use of electric cars may not lead to significant noise reduction effects, especially as the rolling noise related to the moving car (considered the main source of road traffic noise for cars at a speed above 40 km/h [23]) would not be reduced by electric cars. 

Another reason for the marginal well-being effects may be the rather weak association of overall noise perception with subjective well-being which—as in the case of the WHO_5 well-being index—is not based on an evaluative approach but applies multiple items related to both hedonic and eudemonic well-being dimensions. This is likely to reduce the impact that noise—or any environmental feature—may have on the perception of subjective well-being; the application of e.g. noise annoyance as a direct well-being indicator would have probably resulted in a stronger effect. Relevant well-being benefits were therefore only found for the rather small fraction of total population reporting high noise levels at baseline, but did not have a large impact on the total population figures. This is in agreement with other studies suggesting that noise-related well-being changes are more likely to be found in residents strongly affected by noise problems [34,54], and also in line with previous noise studies indicating that self-reported health or well-being may be more related to noise perception than directly to exposure levels [46], suggesting that well-being effects are not linear in relation to noise levels. Yet, it is one of the encouraging findings of this exploratory work that the well-being effect of the transport interventions may be mostly realized in residents most affected by noise.

Lastly, the BAU2020 scenarios may also affect the impact of the Intervention2020 scenarios on probability of well-being. This is documented by the specific case of Basel, which has adopted sustainable urban planning principles many years ago. The Basel BAU2020 thus includes a set of measures reducing transport and related noise emissions (extended tram lines; increased road capacity *etc*.), which already increase the well-being probability for the total population and reduce the impact of additional interventions. The Basel Intervention2020 scenario aimed to reduce traffic on inner roads (where residential density is likely to be lower) and channel more traffic along main roads, where many residents are located and thus is likely to contribute to the modeled increase of high noise levels (13.9% in Intervention2020 *versus* 10.8% in BAU2020) which are associated with a lower well-being probability. 

Residents reporting high levels of noise derive a higher well-being increase, but no overproportional well-being benefit is found in less affluent residents. This suggests that infrastructural interventions to reduce noise can—due to their specific impact on persons severely suffering from noise—help to mitigate noise exposure-related inequalities in well-being. However, such interventions may not be as suitable to reduce social inequalities in noise exposure and address specific target groups. 

### 4.2. Limitations

Although there are some methodological strengths (use of validated survey data assuring comparable data across Europe; enabling coverage of various relevant control variables as covariates, and providing a validated well-being tool in case of EQLS2012 data), a variety of severe methodological limitations and assumptions affect the assessment of well-being impacts based on currently available data. These limitations are discussed below (in chronological order of appearance through the assessment process) and identify the need for further research on well-being effects of urban interventions.

(1) Limitations related to the city noise models:
  - Noise indicators applied
    There are limitations with the noise data applied for the well-being assessments. A general limitation is the use of Lden data which represents the overall noise level during day, evening and night but does not identify peak exposure levels. Although Lden is often used in studies on the long-term impacts of noise exposure and is also suggested as an indicator for noise maps required by the EU Environmental Noise Directive [24], it is unclear whether it is the most suitable noise indicator for well-being impacts.
  - Modelling approaches
    Each city provided their own noise model and methodological differences introduced by local modeling approaches were unavoidable. In addition, non-traffic noise sources were not included in the city models which focused on the detection of transport interventions (see below on the relevance of this limitation).
  - Application of well-being values for 2020 scenarios
    The well-being impact assessment presented has applied noise perception data from EQLS2012 and SHP2012 to derive a predicted well-being probability value for different noise categories. The link between noise perception levels and well-being as derived from EQLS2012 and SHP2012 data is assumed to be applicable for all three scenarios and ignores that traffic noise is expected to be a rising environmental problem in urban settings. Further work would be needed to assess whether the presented approach may lead to over- or underestimation of the well-being impact.
(2) Measurement of well-being

The main limitation refers to the lack of a consistent and undisputed well-being measurement tool and the choices made by our exploratory study to operationalize well-being. Although quite some research is available on this and various well-being tools or scores have been developed [5,13,15,19,20,21], a consensus on the best tool for well-being assessment has yet to emerge. However, based on the existing work, the authors considered the WHO_5 as a pragmatic choice since it (a) was available in the EQLS2012 dataset for many European countries; (b) represents a validated tool based on five items measuring both hedonic and eudemonic well-being dimensions; and (c) already comes with a recommended cut-off to distinguish between low and high well-being. For the Basel case study, the variable considered closest to the WHO_5 well-being index focused more on mental well-being and is thus not fully comparable, indicating the difficulty to identify consistent well-being approaches. In addition there was no default cut-off level to define high *versus* low well-being on the applied response scale from 0‒10.

An alternative approach to well-being measurement would be the application of the concept of “life satisfaction”, which has been recommended by the WHO Regional Office for Europe and the Organisation for Economic Co-operation and Development (OECD) as an international measure of subjective well-being but is based on one question only [13,14]. Comparing EQLS2012 data on life satisfaction with the WHO_5 well-being index suggests that the results tend to be rather similar for Greece (low well-being based on WHO_5: 34.3%; low life satisfaction based on one general question: 30.5%) while there is quite some discrepancy for the Netherlands (20.6% and 8.0%, respectively). This suggests that the respective instruments are not measuring the same concept and work somewhat different in various countries (see Hone *et al*. 2014 [11] and Eurofound/Bertelsmann Stiftung 2014 [60] for the relevance of national variations). While this casts doubt on their comparability, both approaches may be reasonable for application on national or local scale if applied consistently.

(3) Linkage between noise and well-being
  - Use of national proxy data
    Little evidence is available on the link between noise and well-being in general, and specifically on traffic noise and well-being. In the absence of validated risk estimates and due to lack of data on noise-well-being linkages on city level, national EQLS2012 and SHP2012 datasets were used as proxy instead.
  - Use of general noise perception data
    An important issue is that a one-on-one relationship was assumed to exist between noise exposure and noise perception. Ideally, the whole causal chain (from noise exposure through noise perception to subjective well-being) should have been modelled and assessed using empirical data. Lacking such data, the adopted method implicitly assumed that the perception of noise, which also includes noise from neighbours, is determined by the amount of exposure to road traffic noise, which is clearly a simplification. However, the noise perception that can be derived from available surveys covering noise as well as well-being parameters tends to reflect all noise sources but does not specifically ask for perception of separate noise sources. Yet, noise is considered the strongest urban noise source with recent estimates suggesting more than 125 million European citizens in urban areas to be exposed to road traffic noise above 55 dB Lden (the second noise source (railway noise) only affects ca. 16 million European citizens) [23]. Some studies on noise perception exist which allow a relative comparison of various noise sources [34,61,62] but these tend to either report on noise perception only or link it to annoyance or quality of life outcomes. These studies suggest that neighbourhood noise affects general noise perception significantly although its relative contribution is less strong than traffic noise. The European LARES survey on housing conditions and health indicated that neighbourhood noise (32%) is the second relevant noise source after traffic noise (38%) [61], which is in line with a representative German noise perception survey indicating that neighbourhood noise annoyance (33%) is the second-highest noise problem after traffic noise annoyance (56%) in Germany [62]. However, both studies did not report well-being-related impacts of specific noise sources. An Australian study [34] reported that health-related quality of life significantly decreased for citizens reporting high noise annoyance from both road traffic and neighbourhood noise. Yet, based on available evidence, it was not possible to quantify the extent to which overall noise perception is affected by traffic noise *versus* neighbourhood noise and in consequence the impact of this limitation cannot be assessed. This strong restriction will remain a major challenge as long as environmental perception surveys cover noise with one generic question only, failing to separate the most relevant noise sources.
  - Use of cross-sectional data
    In absence of better data, we have used cross-sectional datasets which make it impossible to indicate whether there indeed is a causal relationship between noise and well-being, or whether different levels of well-being possibly affect noise perception. This may especially be the case in Basel, where SHP2012 data on mental well-being was applied which may have affected the rating of noise exposure. However, given that the noise perception data is based on large samples of above 4000 persons, it is likely that the problem of negative/positive affectivity (representing a more negative or positive attitude of a given individual about various aspects such as environmental conditions) would not create a major confounder of the reported noise-well-being relationship as it can be expected to “balance out” within the full sample. This is especially the case because we have used a population based approach and benefit from samples that well represent the general population structure in terms of socioeconomic and demographic features, making a strong over- or underestimation of noise and its influence on well-being rather unlikely. Further limitations of the presented well-being assessment exist which are relevant in methodological terms although they most likely result in an underestimation of well-being benefits.

(4) Exclusive focus on noise effects of transport interventions

The findings presented only indicate the well-being effects of transport-related noise exposure changes attributable to the interventions. The results do not include potential well-being effects triggered through other pathways (e.g., air pollution reduction); such effects are likely to exist and they possibly exceed the well-being effects in relation to noise [54]. Thus, the results presented reflect by no means the total well-being impact of the described urban transport policies.

(5) Restriction of well-being assessment to noise perception categories

Based on the EQLS2012 and SHP2012 data, noise-well-being relationships could only be derived for three (EQLS2012) or two (SHP2012) noise perception categories. The well-being assessment, based on the allocation of different well-being predictor values, only foresees the change of this well-being predictor value for residents that move from one noise perception category (e.g., major problems with noise) to another (e.g., moderate problems with noise). Although noise perception changes are likely to occur for the largest part of the population, they only cause a change of noise perception category for a very small part of the population. Thus, the well-being assessment relies on the change of well-being predictor values for rather few individuals but does not attach a well-being change to individuals which may have benefitted equally from noise reductions but still remain in the same noise perception category. An application of a similar well-being benefit to a larger part of the population would have resulted in significantly stronger results but, based on the available survey data, cannot be documented or calculated. 

Acknowledging the relevance of these limitations in providing a first exploratory well-being impact assessment based on available data, the merit of this paper rather lies in the identification of gaps of evidence and methodology for implementing a more thorough and valid assessment. The quality of the findings reflect the quality of data used and progress on the following aspects is needed to enable more reliable assessments of well-being impacts of urban interventions through:

- Valid, internationally comparable and consensus-based definition and compilation of well-being data;
- Derivation of reliable risk ratios for environmental conditions (such as noise) and well-being;
- Quantification of the relative impact of various noise sources on overall noise perception; and
- Local or national surveys and modeling approaches providing adequate baseline data and enabling the generation of models and future scenarios.

A first step towards establishing a recommended approach towards the measurement of subjective well-being has been taken by WHO Regional Office for Europe with the recent adoption of “life satisfaction” as a core indicator on subjective well-being for monitoring the Health2020 policy of the WHO European Region, aiming at the compilation of consistent well-being data from all European member states [14]. On the impacts of noise on well-being, the forthcoming WHO Environmental Noise Guidelines for the European Region will summarize the existing evidence.

Further research that would move this scientific challenge ahead and enable HIA to also include notions of well-being would especially be longitudinal studies (e.g., taking measures of well-being in a city population before and after an intervention and record changes, also taking into account other factors), or field experiments (e.g., follow up on well-being of e.g., randomly selected residents living in areas highly exposed to traffic *versus* quiet areas and measure well-being over a longer time period).

## 5. Conclusions 

The relation between noise and well-being is not straightforward and requires more study. Dose-response relations have been proposed for noise-related sleep disturbance, annoyance and cardiovascular mortality but not for well-being. Yet, it is accepted that noise is a relevant aspect of well-being and traffic is a primary source of noise. In this work we have critically discussed how the importance of road traffic noise on population well-being can be assessed. The path followed to develop the presented methodology has originated by the lack of specific empirical data at city level and the lack of data on well-being effects of road traffic noise, and is based on a series of decisions and steps that present serious difficulties and limitations. The results therefore need to be interpreted with caution.

The explorative findings from the three case study cities suggest that the noise reduction effects of urban transport interventions are limited but may still trigger slight well-being benefits at population level. The fact that urban transport interventions may yield positive impacts on well-being should not be taken for granted, especially as well-being was not explicitly considered a relevant factor for selection and implementation of the city interventions (which actually focused on climate change mitigation). However, the results also indicate that within a given city population, well-being benefits may be mostly realized within population groups reporting high noise levels, while there is no additional well-being benefit for less affluent population groups when compared with the total population. This could provide an important indication in relation to potential equity effects of urban interventions, but needs to be substantiated by further studies. 

Yet, more importantly, this paper highlights the various methodological constraints and gaps of evidence for exploratory assessments of well-being effects of urban policies. The missing components for a more accurate and valid well-being assessment are discussed and hopefully provoke further research in response to this paper, making well-being assessments increasingly reliable. Especially relevant would be the derivation of validated exposure-response functions for specific environmental determinants and well-being.

## Supplementary Files

Supplementary File 1
