# Supplementary material for: Development of a Quantitative Methodology to Assess the Impacts of Urban Transport Interventions and Related Noise on Well-Being
Source: Int J Environ Res Public Health. 2015 May 26;12(6):5792–814. doi: 10.3390/ijerph120605792 (PMC4483672; doi:10.3390/ijerph120605792)
Supplement: Supplementary File 1 [file ijerph-12-05792-s001.pdf]

Supplementary Information

Development of a Quantitative Methodology to Assess the Impacts of Urban Transport Interventions and Related Noise on Well-Being

Supplementary File 1: Local Noise Data Models and Restrictions of Comparability

The noise data used for the Basel and Rotterdam scenarios was provided by the databases used by the respective local authorities for decision-making in urban planning, while for Thessaloniki the noise modeling has been produced for the URGENCHE project and the model results have been validated against field measurements in two stations (years 2004, 2011 and 2012). Modeling methods differed between the cities as summarized below, but it is not possible to assess to what extent the different approaches may have affected the results.

Basel: The noise exposure model for Basel was provided by the municipality of Basel and takes into account all road traffic (individual motorized traffic as well as tram and bus lines and their frequencies). Noise levels at several façade points were developed using the emission obtained from the local road traffic models and a noise propagation model (CADNA) that link source of emissions to reception points. The model considered building height, first order reflections from building facades, and noise barriers such as e.g., public greenery [1].

Rotterdam: The noise exposure model for Rotterdam only accounts for road traffic noise and excludes other urban noise sources (train, aircraft, trade and industry, neighbourhood). The road traffic noise exposure of the subjects was calculated at the most- and the least exposed facade of the given dwelling with the Dutch standard method SRM2 in accordance with requirements of the EU Environmental Noise Directive (END). The noise calculations are based on road traffic characteristics, including traffic intensity, traffic composition (percentage of light duty, medium duty, and heavy duty vehicles) and speed, and take into account the effects of buildings on propagation of noise.

Thessaloniki: The noise model provided by the Aristotle University of Thessaloniki accounts for road traffic noise covering any road transport. The noise calculations are based on road traffic characteristics (such as traffic intensity) as well as road infrastructure features. Given that the noise impact calculations are clustered at the municipality level, variable residual uncertainty remains around the estimated value on the basis of the urban landscape in each municipality in the Thessaloniki metropolitan area. These uncertainties would tend to slightly underestimate the noise level in 2020.

Supplementary File 2

Table S1. Noise and wellbeing in the case study cities and countries.

Noise and Wellbeing in Switzerland (Source: SHP2012)		
Noise perception	Switzerland, total	Switzerland, urban
Annoyed	20.7%	22.4%
Not annoyed	79.3%	77.6%
Total n	6014	4505

Table S1. *Cont.*

**Noise and Wellbeing in Switzerland (Source: SHP2012)**		
Wellbeing	Switzerland, total	Switzerland, urban
High wellbeing	92.0%	91.8%
Low wellbeing	8.0%	8.2%
Total n	6014	4505
**Noise and wellbeing in the Netherlands (Source: EQLS2012)**		
Noise perception	Netherlands, total	Netherlands, urban
Major problems	1.8%	1.7%
Moderate problems	18.1%	21.0%
No problems	80.2%	77.3%
Total n	1008	582
Wellbeing	Netherlands, total	Netherlands, urban
High wellbeing	79.4%	77.0%
Low wellbeing	20.6%	23.0%
Total n	1005	582
**Noise and wellbeing in Greece (Source: EQLS2012)**		
Noise perception	Greece, total	Greece, urban
Major problems	9.6%	14.4%
Moderate problems	31.6%	40.2%
No problems	58.7%	45.4%
Total n	1003	630
Wellbeing	Greece, total	Greece, urban
High wellbeing	65.7%	64.5%
Low wellbeing	34.3%	35.5%
Total n	1002	629

Supplementary File 3: Coverage of Covariates Potentially Associated With Wellbeing

Several covariates expected to have an effect on wellbeing were included in the EQLS2012 datasets, such as gender, age (5 categories), income (quartiles), education (3 categories), employment (7 categories), making ends meet financially (6 categories) and household structure (5 categories). These covariates were used in their original format, except when the variable values did not provide a clear direction. This was the case for employment (recoded into 3 categories) and household structure (recoded into 4 categories). All other values of the covariate variables were coded "system missing". Within each dataset, the selected covariates were screened for significant bivariate associations with subjective wellbeing using chi-square tests. Significant variables ($p < 0.05$) were then applied as covariates for the binary logistic regression with "good wellbeing" as outcome (see Table below).

For the SHP2012 dataset, similar covariates were available but for household structure and making ends meet financially, some adjustments were necessary: "Household structure" was replaced by "Single household (yes/no)" and "Making ends meet financially" was replaced by "Satisfaction with financial situation (0 (not at all) to 10 (completely satisfied))".

Table S2. Covariates showing significant association with wellbeing in national datasets.

Covariate	Switzerland, Urban	Greece, Urban	Netherlands, Urban
Gender	✓	✓	not significant
Age	✓	✓	not significant
Education	✓	✓	✓
Income	✓	✓	✓
Employment status	✓	✓	✓
Household structure *	✓	not significant	not significant
Making ends meet financially *	✓	✓	✓

* For Switzerland, household structure was replaced by "Single household (yes/no)" and making ends meet financially was replaced by "Satisfaction with financial situation (0 (not at all) to 10 (completely satisfied))".

Supplementary File 4: Overall Prediction of Regression Models

Table S3. Classification Table [a].

Observed		Predicted		
		Wellbeing		
		Low WB	High WB	Percentage Correct
Urban Greece, total sample				
Wellbeing	Low WB	92	65	58.6
	High WB	38	230	85.8
	Overall Percentage			75.8
Urban Greece, less affluent sample				
Wellbeing	Low WB	69	26	72.6
	High WB	26	78	75.0
	Overall Percentage			73.9
Urban Netherlands, total sample				
Wellbeing	Low WB	29	71	29.0
	High WB	17	370	95.6
	Overall Percentage			81.9
Urban Netherlands, less affluent sample				
Wellbeing	Low WB	20	44	31.3
	High WB	6	175	96.7
Overall Percentage				79.6
Urban Switzerland, total sample				
Wellbeing	Low WB	4	364	1.1
	High WB	2	4135	99.9
	Overall Percentage			91.8
Urban Switzerland, less affluent sample				
Wellbeing	Low WB	4	232	1.7
	High WB	2	2015	99.9
	Overall Percentage			89.6

a. The cut value is 0.500.

Supplementary File 5: Establishing Noise Cut-Offs on City Level on the Basis of National Datasets

The matching of the local noise model data and the noise perception categories derived from EQLS2012 and SHP2012 data is explained in the Table below using the example of Thessaloniki.

The EQLS2012 indicated that in Greece, about 14.4% of the urban population report major problems with noise. Transferring these 14.4% to the modeled noise exposure data from Thessaloniki would suggest 65 dB Lden as the most suitable noise cut-off of, as 15.2% of Thessaloniki's population are exposed to 65 dB Lden and beyond. This is the noise level affecting the population percentage closest to 14.4% (cut-off levels at 64 and 66 dB would be less close to the 14.4%). The same approach was applied for moderate and no problems with noise.

Table S4. Derivation of noise cut-offs for the city noise exposure profiles—Thessaloniki example.

Noise Perception	EQLS2012 Data, Urban Greece	Closest Matching Exposure Range for Thessaloniki Noise Model	
Major problems	14.4%	15.2%	≥65 dB Lden
Moderate problems	40.2%	40.6%	55–64 dB Lden
No problems	45.4%	44.2%	≤54 dB Lden
Total n	630 persons	344,244 persons	

The main limitation associated with this approach is that the noise perception data taken from the national surveys results from all noise sources while the noise models provided by the cities reflect only traffic-related noise. Although traffic noise accounts for the largest share of overall urban noise exposure, this approach is therefore problematic and indicates one of the many methodological problems arising for a noise-related wellbeing assessment of urban interventions. Especially unclear is the contribution of neighbourhood noise on the overall perception of noise problems as not many studies provide insight into the relative influence of neighbourhood noise and traffic noise on overall noise perception. This limitation is addressed in more detail in the discussion section of the main paper.

Supplementary File 6: Traffic Noise Changes by City and Scenario

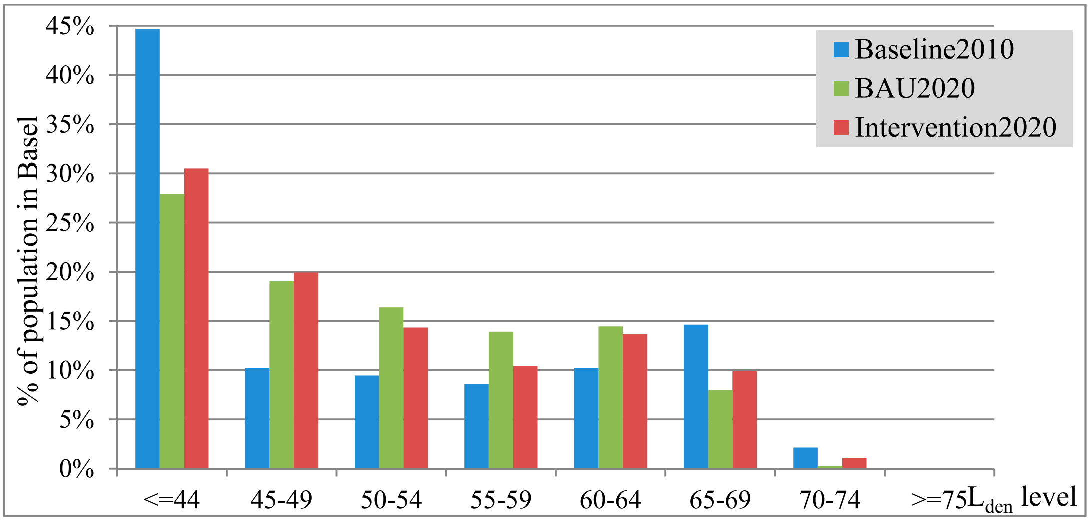

Figure S1. Comparison of traffic noise exposure distribution in the population of Basel at Baseline2010 and under BAU2020 and Intervention2020.

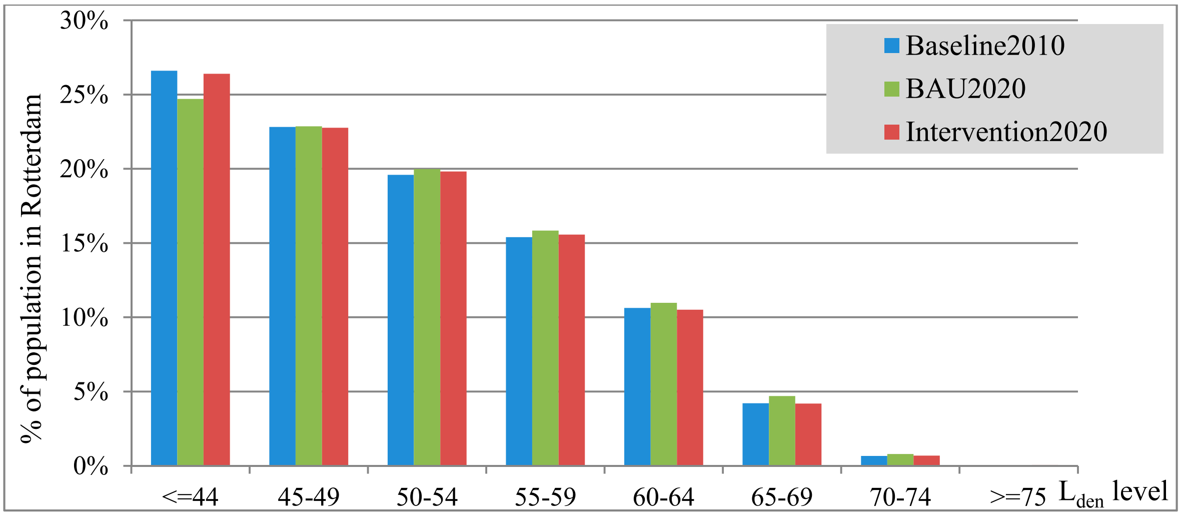

Figure S2. Comparison of traffic noise exposure distribution in the population of Rotterdam at Baseline2010 and under BAU2020 and Intervention2020.

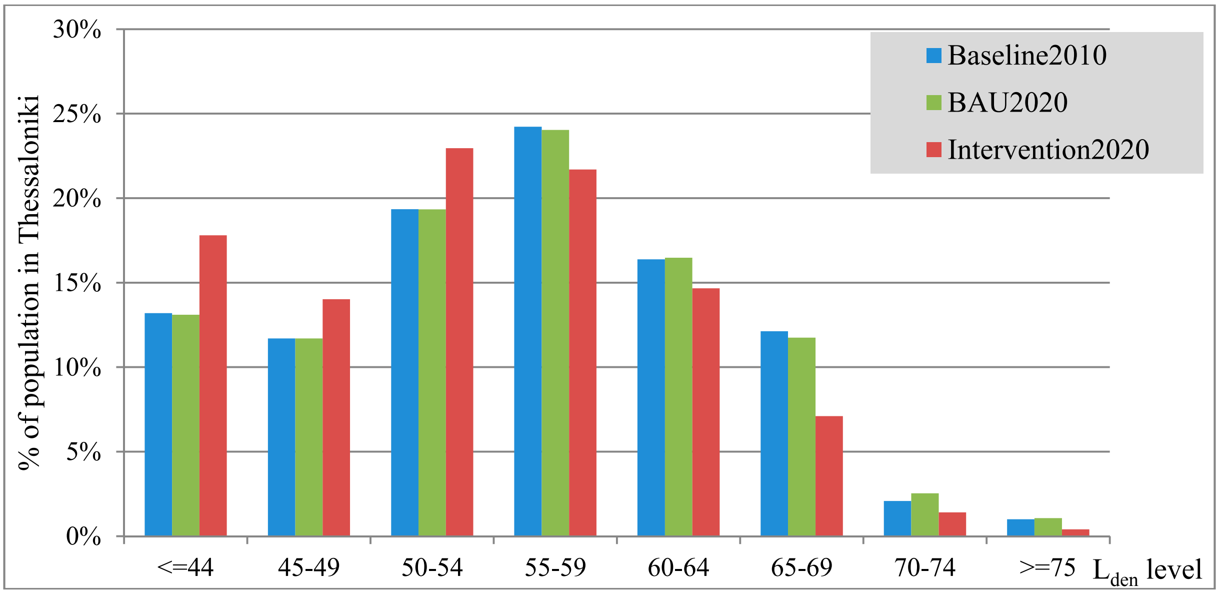

Figure S3. Comparison of traffic noise exposure distribution in the population of Thessaloniki at Baseline2010 and under BAU2020 and Intervention2020.

Supplementary File 7: Detailed Result Tables on Wellbeing Changes in the Less Affluent Population

Table S5. Changes of perceived noise exposure in the less affluent city population.

Level of Perceived Noise Exposure in BASEL	Population Exposed in Baseline2010	Population Exposed in BAU2020	Population Exposed in Intervention2020
	Less Affluent Population	Less Affluent Population	Less Affluent Population
High: Annoyance by noise (≥64 dB)	24.1%	16.9%	19.0%
Low: No annoyance by noise (<64 dB)	75.9%	83.1%	81.0%
**Level of Perceived Noise Exposure in ROTTERDAM**	**Population Exposed in Baseline2010**	**Population Exposed in BAU2020**	**Population Exposed in Intervention2020**
	Less Affluent Population	Less Affluent Population	Less Affluent Population
High: Major noise problem ((≥67.5 dB)	2.5%	2.9%	2.5%
Medium: Moderate noise problem (57.5–67.4 dB)	18.6%	19.5%	18.9%
Low: No noise problem (≤57.4 dB)	78.9%	77.6%	78.6%
**Level of Perceived Noise Exposure in THESSALONIKI**	**Population Exposed in Baseline2010**	**Population Exposed in BAU2020**	**Population Exposed in Intervention2020**
	Less Affluent Population	Less Affluent Population	Less Affluent Population
High: Major noise problem (≥65 dB)	19.9%	20.2%	15.2%
Medium: Moderate noise problem (55–64.9 dB)	39.0%	38.9%	41.4%
Low: No noise problem (≤54.9 dB)	41.1%	40.9%	43.4%

Table S6. Wellbeing probability in relation to noise perception for less affluent population groups.

Level of Perceived Noise Exposure	Predicted Wellbeing Probability (in %)
BASEL	Less affluent urban population sample (n = 2249)
High (≥64 dB)	86.7%
Low (<64 dB)	90.6%
Total population	89.5%
ROTTERDAM	Less affluent urban population sample (n = 245)
High (≥67.5 dB)	67.8%
Medium (57.5–67.4 dB)	70.2%
Low (≤57.4 dB)	74.9%
Total population	73.8%

Table S6. *Cont.*

Level of Perceived Noise Exposure	Predicted Wellbeing Probability (in %)
THESSALONIKI	Less affluent urban population sample (n = 198)
High (≥65 dB)	40.6%
Medium (55–64.9 dB)	56.5%
Low (≤54.9dB)	53.3%
Total population	52.0%

Table S7. Wellbeing probability by noise levels—less affluent population.

Basel	Intervention Implemented by 2020	Predicted Wellbeing Probability (in %)		
		Baseline2010	BAU2020	Intervention2020
Less affluent population	Local transport scenario Z9,	89.5%	90.0%	89.9%
High noise perception	reduction of traffic by 4%	86.7%	87.9%	87.5%
Low noise perception	*(NB: BAU2020 also includes various transport measures)*	90.6%	90.6%	90.6%
**Rotterdam**	**Intervention Implemented by 2020**	**Predicted Wellbeing Probability (in %)**		
		**Baseline2010**	**BAU2020**	**Intervention2020**
Less affluent population		73.8%	73.8%	73.8%
High noise perception	50% of car fleet are	67.8%	67.8%	68.1%
Medium noise perception	electric cars	70.2%	70.2%	70.4%
Low noise perception		74.9%	74.9%	74.9%
**Thessaloniki**	**Intervention Implemented by 2020**	**Predicted Wellbeing Probability (in %)**		
		**Baseline2010**	**BAU2020**	**Intervention2020**
Less affluent population		52.0%	52.0%	52.7%
High noise perception	Local metro built in	40.6%	40.6%	44.6%
Medium noise perception	central Thessaloniki	56.5%	56.5%	56.3%
Low noise perception		53.3%	53.3%	53.3%

References

1. Perez, L.; Trüeb, S.; Cowie, H.; Keuken, M.; Mudu, P.; Ragettli, M. Transport-related measures to mitigate climate change in Basel, Switzerland: A health-effectiveness comparison study. *Environ. Int.* **2015**. (Submitted).

© 2015 by the authors; licensee MDPI, Basel, Switzerland. This article is an open access article distributed under the terms and conditions of the Creative Commons Attribution license (http://creativecommons.org/licenses/by/4.0/).
